# Transcriptomics of early responses to purified *Piscine orthoreovirus*-1 in Atlantic salmon (*Salmo salar* L.) red blood cells compared to non-susceptible cell lines

**DOI:** 10.3389/fimmu.2024.1359552

**Published:** 2024-02-14

**Authors:** Thomais Tsoulia, Arvind Y. M. Sundaram, Stine Braaen, Jorunn B. Jørgensen, Espen Rimstad, Øystein Wessel, Maria K. Dahle

**Affiliations:** ^1^ Departments of Aquatic Animal Health and Analysis and Diagnostics, Norwegian Veterinary Institute, Ås, Norway; ^2^ Department of Biotechnology, Fisheries and Economy, UiT Arctic University of Norway, Tromsø, Norway; ^3^ Department of Medical Genetics, Oslo University Hospital, Oslo, Norway; ^4^ Department of Veterinary Medicine, Norwegian University of Life Sciences, Ås, Norway

**Keywords:** piscine orthoreovirus, red blood cell, Atlantic salmon, salmon kidney cell line, transcriptome

## Abstract

Piscine red blood cells (RBC) are nucleated and have been characterized as mediators of immune responses in addition to their role in gas exchange. Salmonid RBC are major target cells of Piscine orthoreovirus*-*1 (PRV-1), the etiological agent of heart and skeletal muscle inflammation (HSMI) in farmed Atlantic salmon (*Salmo salar*). PRV-1 replicates in RBC *ex vivo*, but no viral amplification has been possible in available A. salmon cell lines. To compare RBC basal transcripts and transcriptional responses to PRV-1 in the early phase of infection with non-susceptible cells, we exposed A. salmon RBC, Atlantic salmon kidney cells (ASK) and Salmon head kidney cells (SHK-1) to PRV-1 for 24 h. The RNA-seq analysis of RBC supported their previous characterization as pluripotent cells, as they expressed a wide repertoire of genes encoding pattern recognition receptors (PRRs), cytokine receptors, and genes implicated in antiviral activities. The comparison of RBC to ASK and SHK-1 revealed immune cell features exclusively expressed in RBC, such as genes involved in chemotactic activity in response to inflammation. Differential expression analysis of RBC exposed to PRV-1 showed 46 significantly induced genes (≥ 2-fold upregulation) linked to the antiviral response pathway, including RNA-specific PRRs and interferon (IFN) response factors. In SHK-1, PRV induced a more potent or faster antiviral response (213 genes induced). ASK cells showed a differential response pattern (12 genes induced, 18 suppressed) less characterized by the dsRNA-induced antiviral pathway. Despite these differences, the RIG-I-like receptor 3 (*RLR3*) in the family of cytosolic dsRNA receptors was significantly induced in all PRV-1 exposed cells. IFN regulatory factor 1 (*IRF1*) was significantly induced in RBC only, in contrast to *IRF3/IRF7* induced in SHK-1. Differences in IRF expression and activity may potentially affect viral propagation.

## 1 Introduction

Red blood cells (RBC) are primarily known for their physiological role in respiratory processes, where intracellular heme and hemoglobin molecules regulate the uptake and transport of oxygen and carbon dioxide (1). In addition to this, a diverse range of physiological and immunologic properties have been attributed to vertebrate RBC, including redox homeostasis, hemoglobin antimicrobial activity and pathogen binding (2, 3). While mammalian RBC are enucleated and lack transcription/translation machinery, teleost RBC have retained their nucleus and organelles in the cytoplasm, essential for intracellular signaling, gene expression and protein production in response to stimuli (2, 4, 5). Previous studies of teleost RBC have shown their ability to react by innate immune responses and physiological differentiation in response to viral infections and systemic signals, respectively (2–4, 6–8). Unlike mammalian RBC, where the nucleus and cellular components are extruded during erythropoiesis to ensure efficient gas exchange (3, 9), transcriptome analyses of teleost RBC has revealed the expression of a complex set of genes involved in virus sensing, antiviral defense and antigen presentation (5, 8, 10, 11). However, the scale of RBC contribution to innate and potentially adaptive immunity is not fully understood.

Viral infections represent a major threat for the piscine aquaculture industry, and efficient prevention remains challenging. Heart and skeletal muscle inflammation (HSMI) is one of the most common viral diseases in farmed Atlantic salmon (*Salmo salar* L.) in Norway (12). The disease is characterized by extensive heart and muscle inflammation with infiltration of immune cells in the epi-, endo- and myocardium, myositis and necrosis in the red skeletal muscle (13–15). The causative agent of HSMI is Piscine orthoreovirus-1 genotype (PRV-1) (14, 16), a member of the order *Reovirales*, family *Spinareoviridae*, genus *Orthoreovirus.* This genus also contains the mammalian and avian orthoreoviruses (MRV and ARV, respectively). PRV-1 has a ten-segmented, double stranded RNA (dsRNA) genome packed in a double-layered icosahedral protein capsid, and was the first orthoreovirus reported in fish (14, 17).

Salmonid RBC are the main target cells of PRV-1 in the primary phase of infection (18). Comparative *in silico* studies with MRV indicate that PRV-1 may use the same infection mechanism, and further studies have indicated that the virus replication occurs in globular neo-organelles referred to as viral factories in the cytoplasm (16, 17, 19, 20). During the peak of infection, high loads of viral RNA and protein are produced within the cells and virus is released into plasma (16, 20). The peak in antiviral responses to PRV-1 has been associated with a decrease in plasma viremia and reduction in viral protein production in RBC (6, 16, 20), along with suppression of some RBC functions, such as hemoglobin production, and expression of metabolic genes (16, 21). Even though the impacts of PRV-1 infection on A. salmon RBC gene expression have been partly characterized *in vivo* and *in vitro* (6, 8, 22), the regulation of genes in RBC shortly after PRV-1 encounter has not been explored in detail.

In the present study, we compared the transcriptomic responses of A. salmon RBC to those of two A. salmon kidney cell lines at resting state, and 24 h after PRV-1 exposure. Atlantic salmon kidney cells (ASK) (23) and Salmon head kidney cells (SHK-1) (24) have been screened and characterized as non-supportive for PRV-1 propagation earlier, showing no evidence of virus replication (25). Here, we report the similarities and differences observed between A. salmon RBC, ASK and SHK-1 before and after PRV-1 exposure, focusing on pathways of the innate immune system.

## 2 Materials and methods

### 2.1 Blood sampling

Six A. salmon pre-smolts (30-50g) were euthanized using benzocaine chloride (1g/5L water) for 5 min, and peripheral blood from the caudal vein was collected in heparinized vacutainers (Vacutest, Sarstedt). The blood was used for isolation of red blood cells.

### 2.2 Isolation of RBC

RBC were isolated from the heparinized blood diluted 1:10 in sterile phosphate buffered saline (dPBS) and laid on top of a Percoll (GE healthcare, Uppsala Sweden) gradient (bottom layer 49%; top layer 34%) which was centrifuged (500 x G, 4°C, 20 min), washed with dPBS and collected as previously described (18). The cells were counted, and their viability was assessed using Countess (Invitrogen, Eugene, Oregon, USA) and resuspended to a concentration of 3 × 10^7^ cells/mL in Leibovitz’s L15 medium (Life Technologies, Carlsbad, CA, USA) supplemented with fetal calf serum (2%) (Sigma- Aldrich) and gentamicin (50 μg/mL- Lonza Biowhittaker, Walkersville, USA). The isolated RBC were inspected by light microscopy in three areas (approximately 100 cells/area, ≥ 300 cells in total) to ensure a maximum of two cells without typical RBC morphology (99% culture purity) (8) The cultures were placed at 15°C under constant agitation (225 rpm).

### 2.3 Atlantic salmon cell line cultures

The A. salmon kidney (ASK) cell line and the Salmon head kidney (SHK-1) cell line, were routinely split (1:2) once a week and cultivated at 20°C in Leibovitz’s L15 medium supplemented with 4 mM L-glutamine (Life Technologies, Carlsbad, CA, USA), fetal bovine serum (10%) (Sigma- Aldrich), 40 μM 2-mecaptoethanol and gentamicin (50 μg/mL- Lonza Biowhittaker, Walkersville, USA). The cells were kept at 15°C during culturing and experiments.

### 2.4 Preparation of purified piscine orthoreovirus-1

Purified PRV-1 was used as inoculum in the *ex vivo* stimulation experiment. The virus was a variant of high virulence (NOR2012) (16), that had been purified from a blood cell pellet of infected fish using cesium chloride density gradient as described previously (16) and stored in Dulbecco’s PBS with 15% glycerol at -80°C. The copy number was determined using absolute quantification RT-qPCR as previously described (16).

### 2.5 *Ex vivo* stimulation

RBC isolated from six fish were plated in NuncTM non-treated 24-well plates with flat bottom (Thermo Fisher) (5 × 10^6^ RBC per well, in 0.5 mL medium). RBC cultures were kept at 15°C under constant agitation (225 rpm) using an Ecotron incubation shaker (Infors HT, Basel Switzerland) to ensure a homogenous suspension. The virus exposure setup included six wells (one per fish) exposed identically to purified PRV-1 (5 x 10^6^ virus particles per well/multiplicity of infection (MOI) of 1) and six control wells (one per fish). Following 24 h of incubation, exposed and control cells were harvested by centrifugation in Eppendorf tubes, media removal and lysis in RT buffer (Qiagen, Hilden, Germany) for RNA isolation.

ASK and SHK-1 experiments were performed at three separate time points (3 parallels). Each time, cells were counted and seeded in 6-well plates with flat bottom (4.5 × 10^4^ cells in 1 mL medium- approx. 80% confluent) (Thermo Fisher) and kept at 15°C in brand incubator. The cultivation setup each time included three wells exposed identically to purified PRV-1 and 3 control wells. Briefly, the cells in the wells were washed three times with dPBS and 4.5 × 10^5^ virus particles (MOI of 10) was added per exposed well. After 24 h of incubation, the cells were washed with dPBS and lysed with RT buffer (Qiagen, Hilden, Germany) for RNA isolation and subsequent RT- qPCR analysis to assess whether PRV-1 was associated with the cells.

### 2.6 RNA isolation and sequencing

Lysed cells were homogenized using 5 mm steel beads and TissueLyser II (Qiagen). Total RNA was extracted using RNeasy Plus Mini Kit (Qiagen, Hilden, Germany) following the manufacturer’s protocol. Isolated RNA was eluted in 50 μL Rnase- free distilled water. RNA was quantified using NanoDrop ND- 1000 spectophotometer (Thermo Fiscer Scientific, Wilmington, DE, USA). RNA quality (RIN >8) was ensured using Agilent 2100 Bioanalyser (Agilent, USA) before being sent for sequencing.

Six biological replicates of the exposed and control RBC (12 samples in total), along with three experimental replicates of the exposed and control kidney cells (6 samples for ASK and 6 samples for SHK-1, respectively) were sent to Norwegian Sequencing Centre (NSC). Library preparation was performed using strand- specific TruSeq RNA Library Prep kit (Illumina, CA, USA). Libraries were subsequently sequenced on Illumina HiSeq to obtain 150 bp paired end reads.

### 2.7 Bioinformatics and statistics

Fastq files of reads from RNA-seq were cleaned (trim/remove adapter and low quality sequences) using BBDuk tool in BBMap v38.22 suite (parameters: ktrim=r, k=23, mink=11, hdist=1, tbo, tpe, qtrim=r, trimq=15, maq=15, minlen=36, forcetrimright=149) (26). Cleaned reads were further mapped to the A. salmon genome (ENSEMBL ICSASG_v2) using the HISAT2 v.2.2.1 (parameters: –rna-strandness RF) (27). FeatureCounts v.1.4.6-p1 (parameters: -p -s 2) was used for estimating the number of reads and aligning against the reference genes in ENSEMBL r104 GTF annotation (28). Initial data analysis was performed using the Bioconductor packages in R, including DESeq2 v.1.34.0 (29) and the SARTools v.1.7.4 (30). Normalization and differential expression analysis were conducted for the cells exposed to the virus against their unexposed controls using DESeq2. The annotation tables were cleaned using median count reads ≥ 10 as a cut off, to get rid of genes with zero or low counts. Subsequently, adjusted p-value (padj) was calculated using Benjamin- Hochberg (BH) correction and gene with padj below 0.05 were considered as differentially expressed genes (DEGs). ShinyGO v0.77 (31) was used for both gene ontology (GO) and Kyoto Encyclopedia of Genes and Genomes (KEGG) enrichment analysis with FDR cutoff 0.05. Pathview R package was used to draw KEGG pathway maps (32, 33).

## 3 Results

### 3.1 Transcriptome analysis of Atlantic salmon RBC and kidney cell lines in resting state

**Figure 1:**
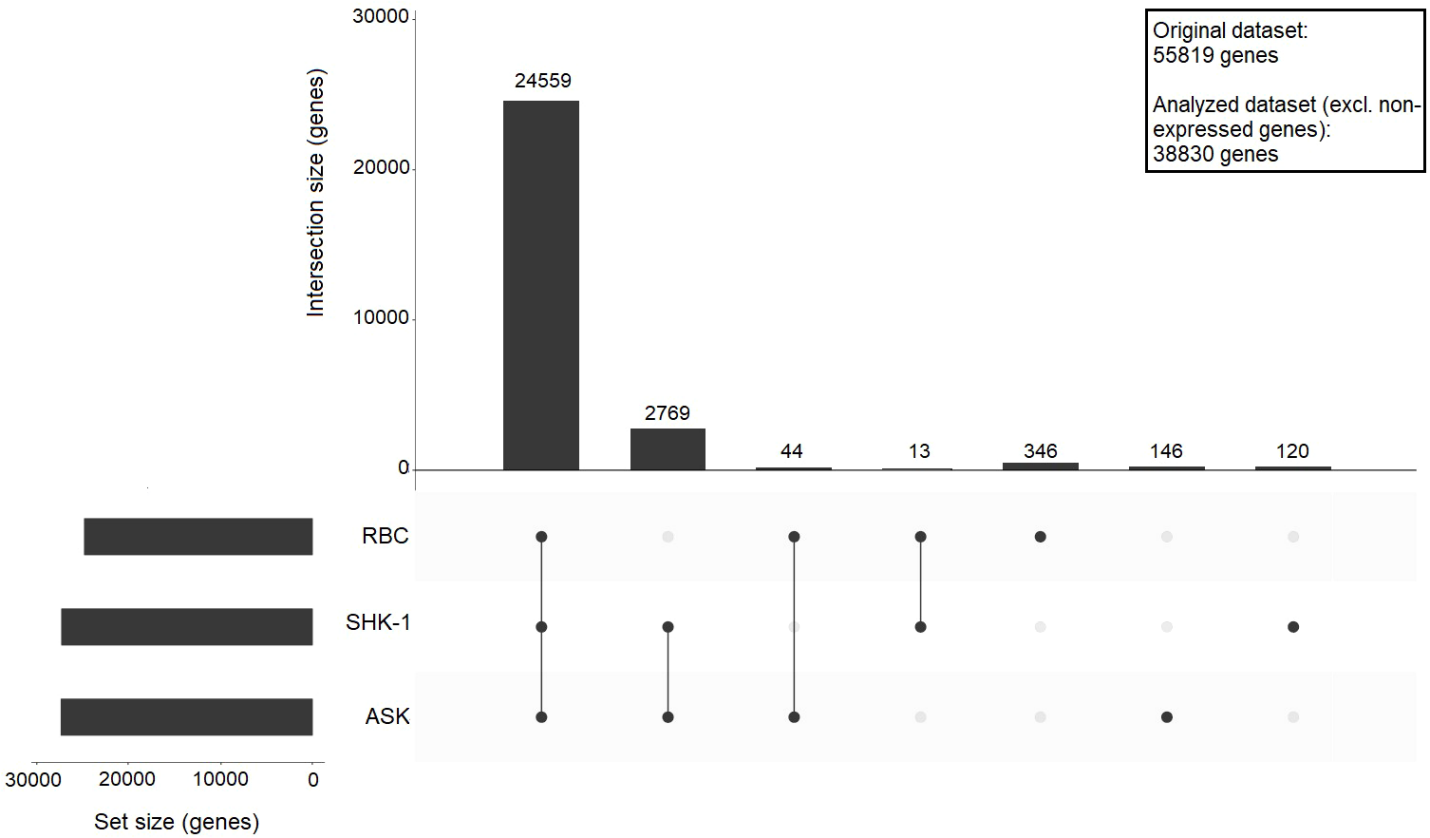
Upset plot showing sharing and unique gene expression for ASK, SHK-1, and RBC. A cutoff ≥ 10 counts was applied to define genes as expressed, and 0 counts required to define genes as not expressed in a cell type; The bars show the number of shared expressed genes between the indicated motifs: RBC vs SHK-1, RBC vs ASK and ASK vs SHK-1, or unique for a specific cell type. The analysis was performed using a dataset of 38830 genes (analysed dataset).

Information on total sequenced reads and alignment rate of mapping of all biological conditions is provided in **Supplementary File A, Table 1**. Normalized RNA- seq data were compared to identify features that are differentially expressed between RBC and kidney cell lines, ASK and SHK-1, at the unexposed resting state. The variability of the biological conditions within the experiment was assessed with a principal component analysis (PCA) (**Supplementary File A, Figure 1**). This analysis showed low variability within the biological (RBC) and experimental (ASK, SHK-1) replicates of each cell type, confirming consistency in the data, while the distribution of the clusters against the two first principal components indicated that SHK-1 and ASK are more closely related.

### 3.2 Transcriptional profiling of Atlantic salmon RBC and kidney cell lines, ASK and SHK-1

The original dataset consisted of 55819 features (genes). After filtering out 16989 genes with zero normalized median count reads, the differences and similarities in the expression profile of RBC, ASK and SHK-1 were assessed using an upset plot, including 38830 features (referred to as analyzed dataset) (**Figure 1**). A cutoff ≥ 10 counts was applied, and 24962 genes were found transcribed in RBC, 27518 genes in ASK and 27461 in SHK-1. In the three cell types, 24559 common genes were expressed. ASK and SHK-1 were sharing 2769 expressed genes (ASK & SHK-1 cutoff ≥ 10 median counts, RBC = 0 median counts), verifying their highest level of similarity as indicated by PCA. A subset of 346 genes were exclusively expressed in RBC, while 44 genes were only expressed in RBC and ASK, and 13 genes were only expressed in RBC and SHK-1 (**Figure 1**).

To identify the processes in which the genes of each subset are involved, Kyoto Encyclopedia of Genes and Genomes (KEGG) pathway enrichment analysis was performed. The lists of gene functional groups found in the enrichment analysis are provided in **Supplementary File B**. RBC, ASK and SHK-1 appeared to all share genes related to fundamental cellular processes, such as endocytosis, protein processing in ER and ubiquitin mediated proteolysis. Two KEGG pathways associated to cellular responses activated by viral and bacterial invasion, “Herpes simplex virus 1 infection” and “Salmonella infection” respectively, showed the greatest representation of shared genes (456 and 410 genes, respectively) between RBC, ASK and SHK-1. This indicated that RBC possess immune functions similar to ASK and SHK-1 and are able to respond to viral and bacterial pathogens. The KEGG pathways named “Herpes simplex virus 1 infection” and “Salmonella infection” were first described in mammals in response to these pathogens but have also been identified in teleost (33). In this study, the official KEGG nomenclature is used even if they refer to pathogens not relevant for this study.

#### 3.2.1 Gene ontology enrichment analyses for the genes exclusively mapped to RBC

The subset of genes mapped exclusively in RBC consisted of 346 features. To identify biological processes that may be regulated by these genes, gene ontology (GO) enrichment analysis on Biological Process (GO : BP) was performed. Most genes were involved in “Cell surface-” and “G protein-coupled receptor” signaling pathways, whereas only a few appeared to contribute to physiological processes, such as gas transport and respiratory burst. Regarding the immune characteristics of the cells, genes involved in chemotaxis (e.g. C-C chemokine receptor type 9 (*CCR9*) and C-C motif chemokine 4 (*CCL4*) –like), phagocytosis (e.g. coronin-1A-like) and innate immune response pathway [e.g. interferon regulatory factor 4 (*IRF4*) and interleukin-1 receptor type II (*IL1R2*)] were represented. The detailed GO : BP categories along with the list of the 346 genes are provided in **Supplementary File B**. KEGG pathway enrichment analysis was considered inconclusive for such a small input.

### 3.3 Identification of differentially expressed genes between Atlantic salmon RBC and kidney cell lines, ASK and SHK-1

**Figure 2:**
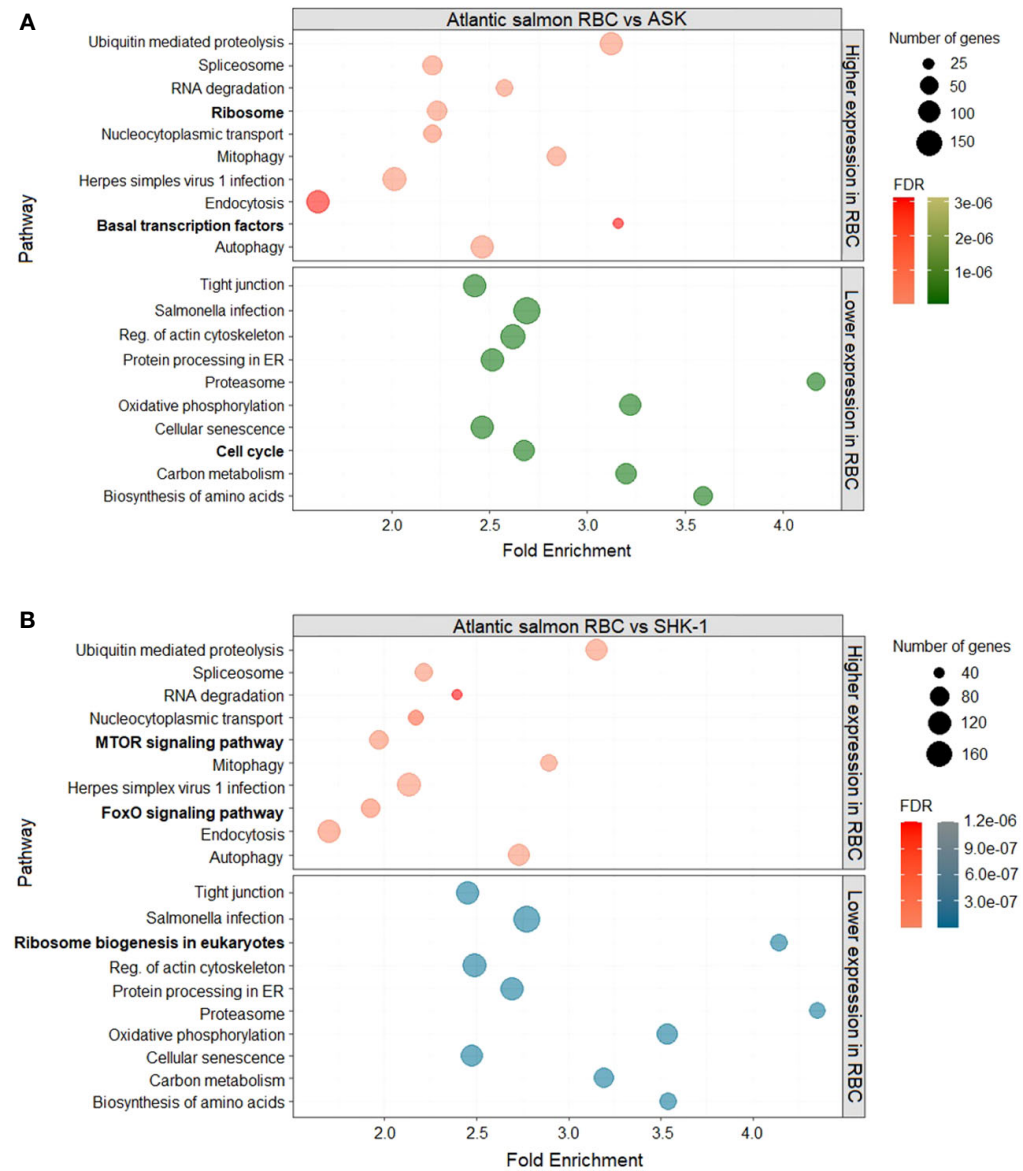
DEGs of RBC (red) compared to the kidney cell lines, **(A)** ASK (green) and **(B)** SHK-1 (blue). Kyoto Encyclopedia of Genes and Genomes (KEGG) pathway enrichment analysis was further analysed in ShinyGO 0.76 for FDR cutoff ≤ 0.05 and DEGs with fold-change ≥ 2 and ≤ 0.5.

Differential gene expression analysis was performed to estimate differences in gene expression patterns between RBC and each kidney cell line (ASK and SHK-1). Filtering out low count genes (cutoff ≥ 10 median counts), the comparison of RBC against ASK and SHK-1 resulted in 14493 and 14397 differentially expressed genes (DEGs), respectively (**Supplementary File A, Figure 2**). In both comparisons, approximately 7500 DEGs indicated higher expression levels in RBC (thus, lower expression levels in ASK and SHK-1). Accordingly, approximately 6800 DEGs indicated lower expression level in RBC (thus, higher expression levels in ASK and SHK-1). ASK vs SHK-1 resulted in 10018 DEGs, 5041 with higher expression levels in SHK-1 and 4977 with higher expression level in ASK. The lists of DEGs emerging from the comparison of RBC vs SHK-1, RBC vs ASK and ASK vs SHK-1 are provided in **Supplementary File C**.

**Figure 3:**
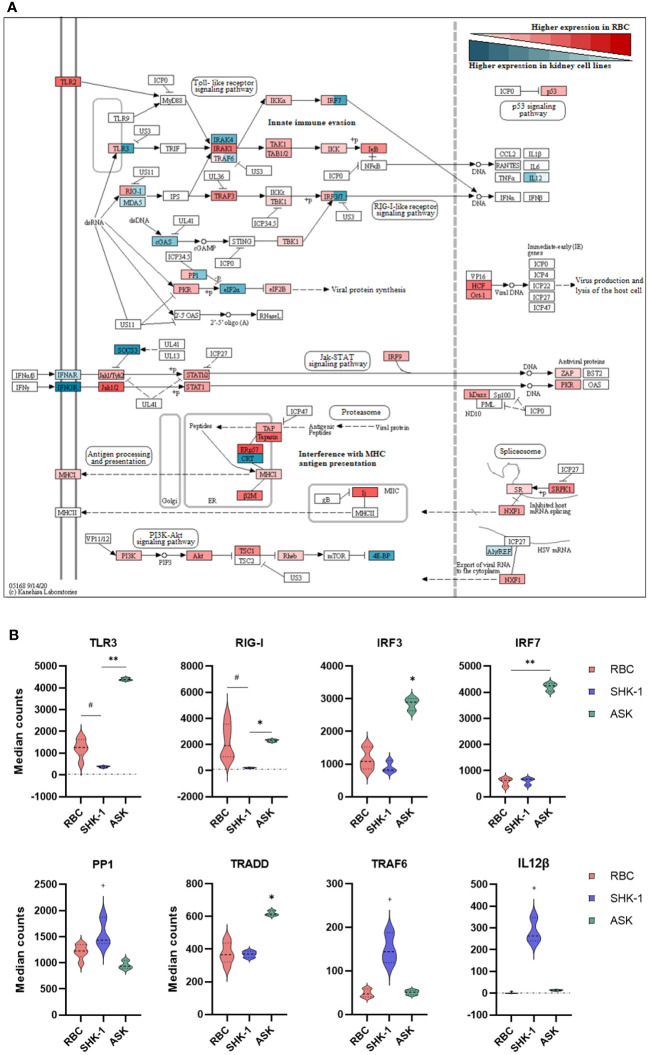
Differential expression analysis of selected genes associated with innate immunity in RBC, ASK and SHK-1. **(A)** Signaling pathways triggered by viral invasion. Red: Significantly higher normalized counts in RBC; Cyan: Similar and Significantly higher normalized counts in ASK and SHK-1. Red and cyan: Significantly different expression levels between ASK and SHK-1, and also with RBC were colored in both red and cyan. This figure was modified from the “Herpes simplex virus 1 infection” pathway- sasa05168 in KEGG, to include only immune pathways of interest. **(B)** Selected genes with significantly different expression pattern between the kidney cell lines, ASK and SHK-1, and RBC. ^#^p ≤ 0.05 in RBC vs ASK and SHK-1; *p ≤ 0.05 in ASK vs RBC and SHK-1; **p ≤ 0.05 in ASK vs RBC and SHK-1; ^+^p ≤ 0.05 in SHK-1 vs ASK and RBC.

To determine the pathways to which DEGs of RBC vs ASK and SHK-1 belonged, KEGG pathway enrichment analysis was performed. The analysis was performed for DEGs with normalized median counts ≥ 10 and fold- change ≤ 0.5 for the downregulated genes in ASK and SHK-1 compared to RBC (i.e “Higher expression compared to RBC” group of genes) and ≥ 2 for the upregulated genes in ASK and SHK-1 compared to RBC (i.e"Lower expression compared to RBC" group of genes). The majority of DEGs with higher expression in RBC compared to both ASK and SHK-1 were involved in innate immune processes related to viral sensing (KEGG nomenclature “Herpes simplex virus 1 infection”) (119 and 126 genes, respectively), as shown in **Figures 2**, **3** in detail. Several genes with significantly higher transcripts in RBC were also involved in pathways associated with cellular functions like “Endocytosis”, “Autophagy” and “Ubiquitin mediated proteolysis” (**Figure 2**). RBC DEGs belonging to KEGG groups, “MTOR-” and “FoXO” signaling pathways were only reported in the comparison of RBC vs ASK (**Figure 2A**, top), while “Ribosome” and “Basal transcription factors” in RBC vs SHK-1 (**Figure 2B**, top).

The majority of DEGs with lower expression in RBC were primarily involved in processes of cytoskeleton and paracellular communication (“Reg. of actin cytoskeleton” and “Tight junction”) and host defense against bacterial invasion (“Salmonella infection”). Several genes were grouped within KEGG categories related to cellular senescence, metabolism and oxidative phosphorylation (**Figure 2**). Genes involved in ribosome biogenesis were more highly expressed in SHK-1 compared to RBC, indicating that RBC are less active in protein production (**Figure 2B**, bottom). Results from RBC vs ASK showed that genes linked to cell cycle events were more highly expressed in ASK (**Figure 2A**, bottom), which is expected for a continuous cell line.

To better understand the role of RBC in modulating functions of the innate immune system, we focused on signaling pathways involved in viral sensing and infection. These are included in the KEGG category referred to as “Herpes simplex virus 1 infection-sasa05168” pathway that consisted of the largest amount of DEGs with significantly higher expression levels in RBC. **Figure 3A** was extracted from the original pathway sasa05168 as established by Kanehisa Laboratories (2020). The detailed modified pathway is provided in **Supplementary File A, Figure 3**.

RBC expressed genes involved in toll-like receptor (TLR) and RIG-I-like receptor (RLR) signaling. Several signaling mediators in these pathways, such as interleukin 1 receptor associated kinase 1 (*IRAK1*) and TNF receptor associated factor 3 (*TRAF3*), showed a higher expression level in RBC compared to ASK and SHK-1 (**Figure 3A**). However, the basal expression levels of pattern recognition receptors (PRRs), *TLR3*, melanoma differentiation-associated protein 5 (*MDA5*) and *RLR1* (also referred to as *RIG-I or DDX58)*, and interferon regulatory factors (*IRF*) 3 and 7 were significantly higher in ASK. Several components essential to antigen processing and presentation (MHCI pathway), inhibition of viral production (*PKR* regulation and *Jak-STAT* signaling pathway) and regulation of apoptosis and viral propagation (*PI3K- Akt* pathway) showed significantly higher transcripts in RBC than ASK and SHK-1 (**Figure 3A**). While ASK and SHK-1 indicated similar expression patterns overall, a few genes related to cell cycle and immune cell differentiation were expressed significantly higher in SHK-1 (**Figure 3B**).

### 3.4 Identification of innate immune function genes in Atlantic salmon RBC

RBC have traditionally been characterized exclusively as gas exchangers expressing hemoglobins (3). As expected, several hemoglobin (Hb) subunits were found among the most highly expressed genes in RBC in the dataset (**Table 1**), also indicating culture purity. Expression levels of iron storage ferritins and mediators of heme biosynthetic pathway (such as *BLVRB* and *ALAS2*), which typically function in blood/RBC (34, 35), were also among the highest expressed genes. Also, MHC class I- related gene protein-like and thymus-specific serine protease (*TSSP*) antigen processing components were among the most highly expressed genes in salmonid RBC (**Table 1**). To further assess the purity of the RBC culture, transcripts of typical T cells and B cells markers were sought and evaluated. While many were not identified in our datasets, such as *CD3* and *CD34*, a few typical T cell and B cell markers such as *CD4* and *CD8* (36), showed near- zero count reads (**Table 1**).

**Table 1:** Transcript counts of the 20 most highly expressed genes in A. salmon RBC compared to ASK and SHK-1.

Gene	Description	Ensembl ID	RBCs (counts)	ASK (counts)	SHK-1 (counts)
*HBAA2*	Hemoglobin subunit alpha-4	*ENSSSAG00000044737*	797987	157	172
*-*	Ferritin heavy subunit	*ENSSSAG00000049977*	671668	112424	70642
*HBB1*	Hemoglobin subunit beta-1-like	*ENSSSAG00000044957*	579951	130	137
*-*	Hyperosmotic glycine rich protein	*ENSSSAG00000068063*	421881	200118	163430
*HBA4*	Hemoglobin subunit alpha-4	*ENSSSAG00000065254*	321654	89	87
*HBB*	Hemoglobin subunit beta-like	*ENSSSAG00000045065*	321344	83	92
*HBA*	Hemoglobin subunit alpha	*ENSSSAG00000065229*	244043	75	68
*HSPA8*	Heat shock protein 8	*ENSSSAG00000049191*	213336	55203	37017
*HBB*	Beta globin	*ENSSSAG00000065233*	210828	44	47
*HBB1*	Hemoglobin subunit beta-1-like	*ENSSSAG00000065315*	187925	106	117
*FRIH*	Ferritin, heavy polypeptide 1-1	*ENSSSAG00000051567*	156074	32006	99410
*HBBA2*	Hemoglobin subunit beta-1-like	*ENSSSAG00000065226*	150808	45	44
*NRK2*	Nicotinamide riboside kinase 2-like	*ENSSSAG00000077245*	142822	9601	2613
*TSSP*	Thymus-specific serine protease	*ENSSSAG00000053130*	136772	32	38
*BLVRB*	Biliverdin reductase B	*ENSSSAG00000069097*	117596	965	1314
*ALAS2*	5’-aminolevulinate synthase 2	*ENSSSAG00000068428*	106223	28	30
*-*	Major histocompatibility complex class I-related gene protein isof. X1	*ENSSSAG00000077419*	87427	29250	54093
*MIBP2*	Nicotinamide riboside kinase 2-like	*ENSSSAG00000068654*	79622	4905	3683
*WBP4-like*	WW domain-binding protein 4-like	*ENSSSAG00000077000*	78270	434	278
*5NTC*	Cytosolic purine 5-nucleotidase	*ENSSSAG00000045618*	67967	23	21
***Cd4***	***S. salar* T-cell surface glycoprotein CD4**	***ENSSSAG00000076595***	**1**	**6**	**0**
***Cd8a***	***CD8- alpha***	***ENSSSAG00000065860***	**0**	**0**	**0**
***Cd8b***	***CD8- beta***	***ENSSSAG00000045680***	**1**	**0**	**0**
***Cd34***	***CD34* molecule**	***ENSSSAG00000079346***	**0**	**952**	**589**
***MME***	**Neprilysin- like**	***ENSSSAG00000042374***	**5**	**0**	**1**

Transcript counts of five distinct T cells and B cells markers (in bold) were also included to assess RBC culture purity. The expression levels of the genes were measured as median normalized count reads (counts). All listed genes indicated significantly higher expression in RBC (p ≤ 0.5).

To assess the contribution of RBC to innate immunity, we focused on identifying components associated with pathogen recognition, cell-to-cell communication, activation of the innate immune system and host defense. The detection of infectious agents is mainly mediated by (germline-encoded) PRRs. PRRs are highly conserved among vertebrates and the main families described in fish include toll-like receptors (TLRs), nucleotide oligomerization domains (NOD) -like receptors, retinoid acid-inducible (RIG) -like receptors (RLRs), C-type lectin receptors (CLRs) and scavenger receptors (SRs) (37). A wide repertoire of PRRs from all five families was found in RBC. TLRs, RLRs and NLRs were the most abundant PRRs in the cells and those with the highest transcript levels are listed in **Table 2**. RLRs, which primarily recognize double- stranded (ds) RNA oligonucleotides, showed collectively the highest expression. *TLR3*, previously identified in salmonid RBC and known to bind dsRNA, was detected in high transcript numbers (8). *TLR8*, which recognizes single- stranded (ss) RNA, showed the highest expression among the TLRs (38, 39). Several NLRs, which primarily have been characterized in mammals as sensors of bacterial components, such as lipopolysaccharides (LPS) and peptidoglycans (PGNs) were identified in RBC. Variants of NLR family CARD domain containing 3- like (*NLRC3L*) showed the highest expression (45). In addition, *NLRC5* and *NOD1/NOD2* were detected. Their role and functionality in teleosts are modestly studied.

**Table 2:** Pattern recognition receptors (PRRs) identified in A. salmon RBC.

Gene	Ensembl ID	RBC (counts)	Ligands	Reference in teleost
Toll-like receptors (TLRs)				
TMSB4X (or TLR8)	*ENSSSAG00000076485*	2060	ssRNA	(38)
*TLR3*	*ENSSSAG00000040910*	1244	dsRNA	(8)
*TLR2*	*ENSSSAG00000003781*	50	LPS	(39)
*TLR19*	*ENSSSAG00000042328*	31	Non specified	(39)
Retinoic acid-inducible gene (RIG)-like receptors (RLRs)				
*MDA5*	*ENSSSAG00000078885*	2264	dsRNA	(40)
*DDX58*	*ENSSSAG00000045391*	2232	(ds)RNA	(41)
*DHX58*	*ENSSSAG00000037858*	1824	ssRNA; dsRNA	(40)
Nucleotide oligomerization domains (NOD)-like receptors (NLRs)				
*NLRC3L1*	*ENSSSAG00000005336*	1461	DNA and RNA oligonucleotides	(42)
	*ENSSSAG00000056446*	1177		
	*ENSSSAG00000046213*	1033		
*NLRC5*	*ENSSSAG00000068298*	233	Bacterial components	(43)
*NOD1*	*ENSSSAG00000053537*	170	Bacterial PGNs	(44)
*NOD2*	*ENSSSAG00000076025*	26	Bacterial PGNs	(44)

The majority of mapped PRRs were categorized in 3 major groups: toll-like receptors (TLRs), retinoic acid inducible gene (RIG)- like receptors and nucleotide- oligomerization domain (NOD)- like receptors. The basal expression levels of the genes were measured as median normalized count reads (counts). Only genes with transcripts ≥ 10 (cutoff ≥ 10 median counts) were included in the analysis. LPS, lipopolysaccharides; PGNs, peptidoglycans.

**Figure 4:**
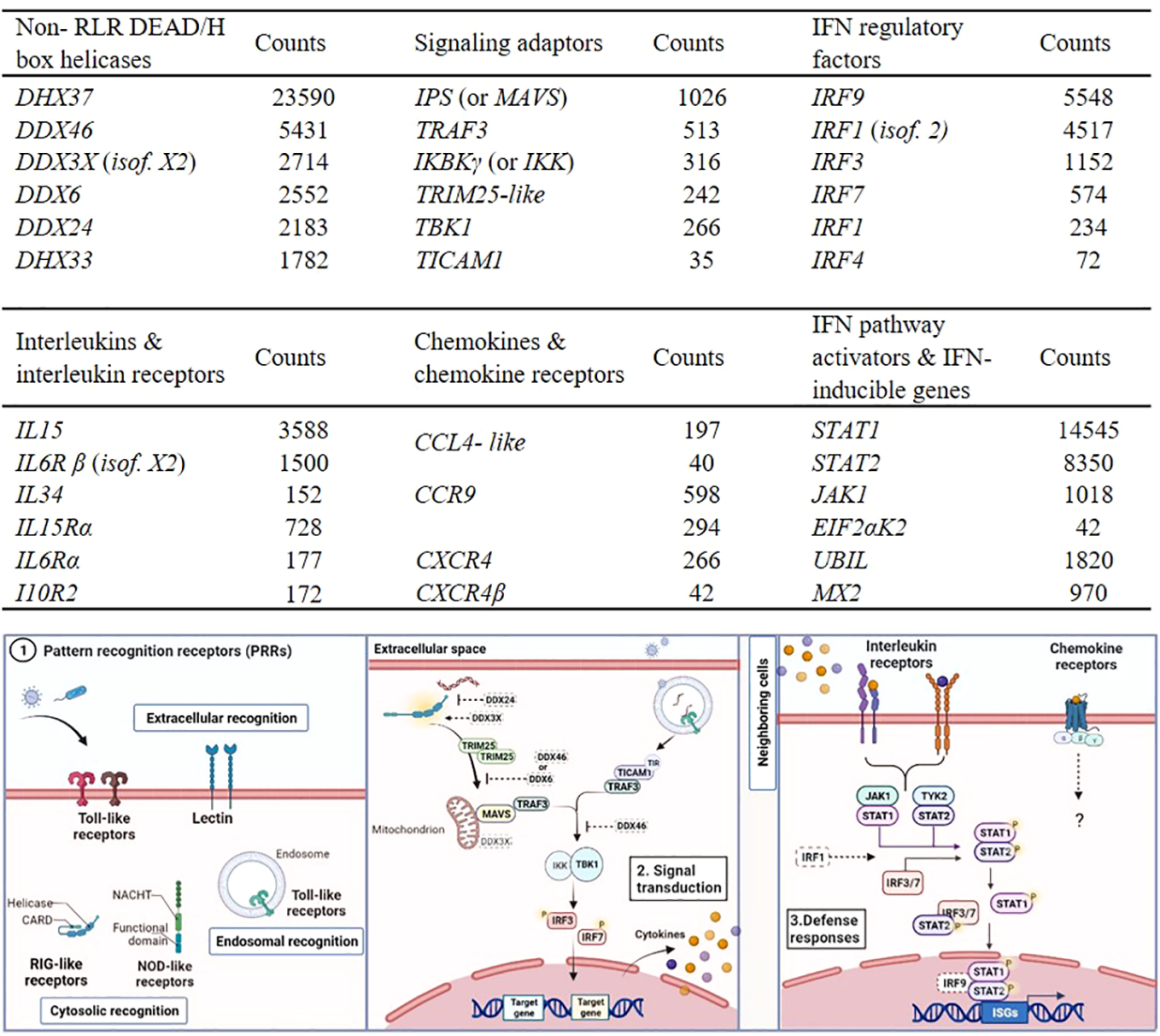
Examples of RBC genes involved in innate immune responses identified in A. salmon RBC. Transcripts of non- RLR DEAD/DEAH box helicases, signaling adaptors and interferon regulatory factors (IRFs) (table on top). Transcripts of interleukins (ILs) and interleukin receptors (ILRs), chemokines (C-C and C-X-C motifs) and chemokine receptors and interferon (IFN) pathway activators and IFN- inducible genes (table on bottom). The expression levels of the genes were measured as median normalized count reads (counts) (RBC n= 6). Only genes with transcript reads ≥ 10 (cutoff ≥ 10 median counts) were included in the analysis. Short description of the pathways relevant for genes expressed in RBC and listed in the tables above. Elements drawn in dash have not been characterized in teleost, and their roles were based on mammalian models. Step 1. Pattern recognition receptors (PRRs). Step 2. Signaling mediators and interferon regulatory factors acting downstream of PRR binding, leading to secretion of IFNs and pro-inflammatory cytokines. Step 3. Pathways induced when secreted IFNs and cytokines bind to receptors, leading to expression of several innate immune effectors.

The majority of the signaling regulators and effectors which interact with TLRs and RLRs, along with various non-RLR DEAD/DEAH box RNA helicases with diverse roles in innate immunity, were identified in RBC, as shown in **Figure 4** (top). Indicatively, *DHX37* showed the highest expression level, however details about its function have not been determined in either fish or mammals. *IRF1* (isoform 2), known to regulate the induction of interferon (*IFN*) and IFN-stimulated genes, and *IRF9*, associated with antiviral immunity (46), were highly expressed in the RBC transcriptome. Several cytokine receptors were found in our dataset, but only a few cytokines (interleukins and chemokines) were expressed in RBC, including interleukin 15 and 34 (*IL15* and *IL34*), and *CCL4- like* chemokine (**Figure 4**). Common IFN stimulated antiviral effector genes, such as IFN stimulated gene 15 (*ISG15*) like (*UBIL*) and myxovirus resistance (*Mx2*), known to be induced by IFNs, were also identified in RBC in high transcript numbers.

### 3.5 Differential expression analysis of RBC and kidney cell lines exposed to PRV-1

**Figure 5:**
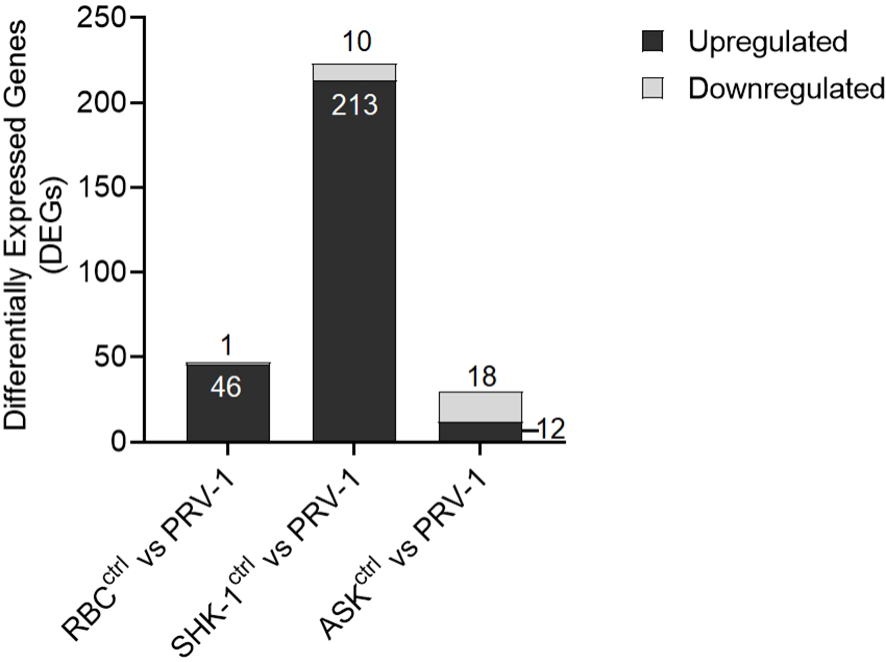
Differential gene expression analysis of A. salmon RBC, SHK-1 and ASK exposed to PRV-1 for 24h compared to their unexposed controls (RBC vs PRV-1, SHK-1 vs PRV-1 and ASK vs PRV-1, respectively). The analysis was performed on genes with median counts ≥ 10. Cutoff ≥ 2-fold change for upregulated DEGs and ≤ 0.5-fold change for downregulated DEGs was applied.

To identify the antiviral responses in RBC at early PRV-1 exposure (24 h) compared to non- susceptible cell lines, normalized RNA-seq data of the samples exposed to the virus were compared to unexposed controls through differential expression analysis (DESeq2). Information on total sequenced reads and alignment rate of mapping, along with principal component analysis (PCA) are provided in **Supplementary File A, Figure 4**. Differential expression analysis of RBC exposed to PRV-1 vs the unexposed controls showed a set of 46 significantly induced genes (≥ 2-fold upregulation) and 1 significantly suppressed (≤ 0.5-fold downregulation) gene (**Figure 5**). In contrast, 213 genes were significantly induced and 10 genes were significantly suppressed in SHK-1. In ASK, 12 genes were significantly induced and 18 genes significantly suppressed. Thus, SHK-1 demonstrated the strongest and ASK the weakest responses to PRV-1.

### 3.6 GO and KEGG enrichment analysis for the DEGs of RBC, ASK and SHK-1 exposed to PRV-1

**Figure 6:**
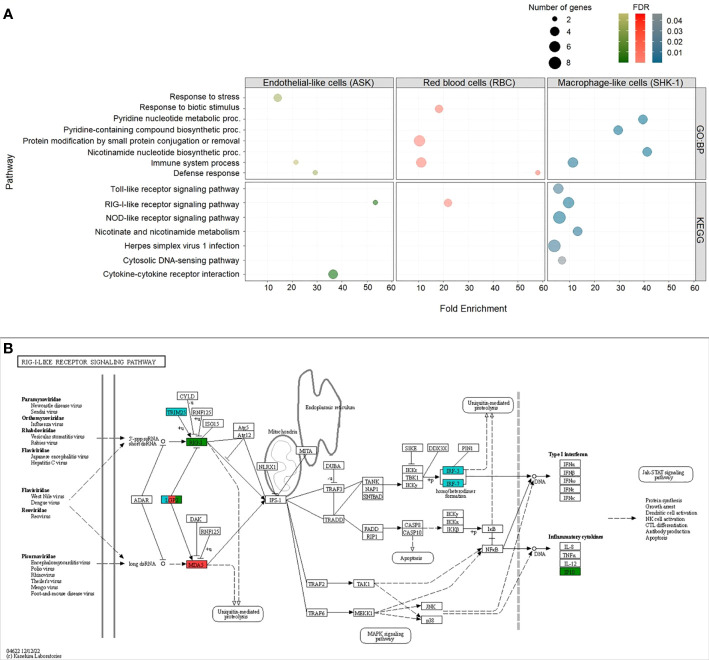
Up-regulated DEGs (cutoff ≥ 2-fold change) in ASK, RBC and SHK-1 exposed to PRV-1. Enriched Gene Ontology (GO) terms within the GO category “Biological Process” (GO : BP) and Kyoto Encyclopedia of Genes and Genomes (KEGG) pathways with FDR (adjusted p value) lower than 0.05 were considered significant. **(A)** GO : BP (top) and KEGG pathways (bottom) enriched in ASK (green), RBC (red) and SHK-1 (blue). **(B)** Representation of KEGG pathway:”RIG-I-like receptor signaling pathway”-sasa04622, as significantly enriched in RBC, ASK and SHK-1. Genes involved in pathway and significantly induced in RBC, ASK and SHK-1 exposed to PRV-1 were annotated in red, green and blue, respectively.

We performed GO and KEGG pathway enrichment analyses with an FDR (adjusted p value) cutoff of 0.05 for the upregulated DEGs (≥ 2-fold change) in RBC, ASK and SHK-1 to identify biological processes and signaling pathways activated in response to PRV-1 (**Figure 6**). As the significantly downregulated genes were too few, they were not subjected to these analyses. GO enrichment analysis for Biological Process (GO : BP) resulted in 9 GO terms for RBC, 6 for SHK-1 and 3 for ASK. Genes in RBC were mainly involved in four biological processes: “Response to biotic stimulus”, “Protein modification by small protein conjugation or removal”, “Defense response” and “Immune system process”. GO term “Immune system process” consisted of 6 genes, including *RLR3* [also referred to as laboratory of genetics and physiology 2 (*LGP2*)], melanoma differentiation-associated protein 5 (*MDA5*) and transcription factors involved in *type I IFN*-pathway activation, *IRF1-2* and *IRF1*. From the GO terms that appeared for ASK, biological functions associated with response to stress showed the greatest representation, while groups “Immune system process” and “Defense response” consisted of only two significantly expressed genes, one of which was *RLR3*. Other significantly induced genes in SHK-1 were primarily involved in metabolic functions associated with the formation of nicotinamide-adenine dinucleotide phosphate, such as “Pyridine nucleotide metabolic process”, “Pyridine-containing compound” and “Nicotinamide nucleotide” biosynthetic processes. The GO : BP term “Immune system process” was also significantly enriched for SHK-1, including genes such as the dsRNA receptors *RLR3* and *TLR3*, and the antiviral effectors, *UBIL* and *Mx2*. A detailed description of GO terms in RBC, ASK and SHK-1 is provided in **Supplementary File D**.

KEGG analysis revealed one category, “RIG-I-like receptor signaling pathway”- sasa04622, which was significantly enriched in RBC, ASK and SHK-1. This category consists of genes involved in immune pathways activated upon binding of dsRNA to RLRs, including the *RLR3* gene (referred to as *LGP2* in the pathway). The cytosolic dsRNA receptor *MDA5* gene was induced only in RBC, and the *RLR1* gene was induced only in ASK (**Figure 6B**). In SHK-1, the tripartite motif-containing protein 25 (*TRIM25*) gene, *IRF3* and *IRF7* in this pathway was also significantly induced (**Figure 6B**). In contrast to RBC, genes significantly induced in SHK-1 were categorized in five more groups, four of which are involved in innate immunity (such as “Toll like receptor” and “NOD-like receptor” signaling pathways), while significantly induced genes in ASK were categorized in one additional group, associated with cytokine- cytokine interaction (**Figure 6A**).

**Figure 7:**
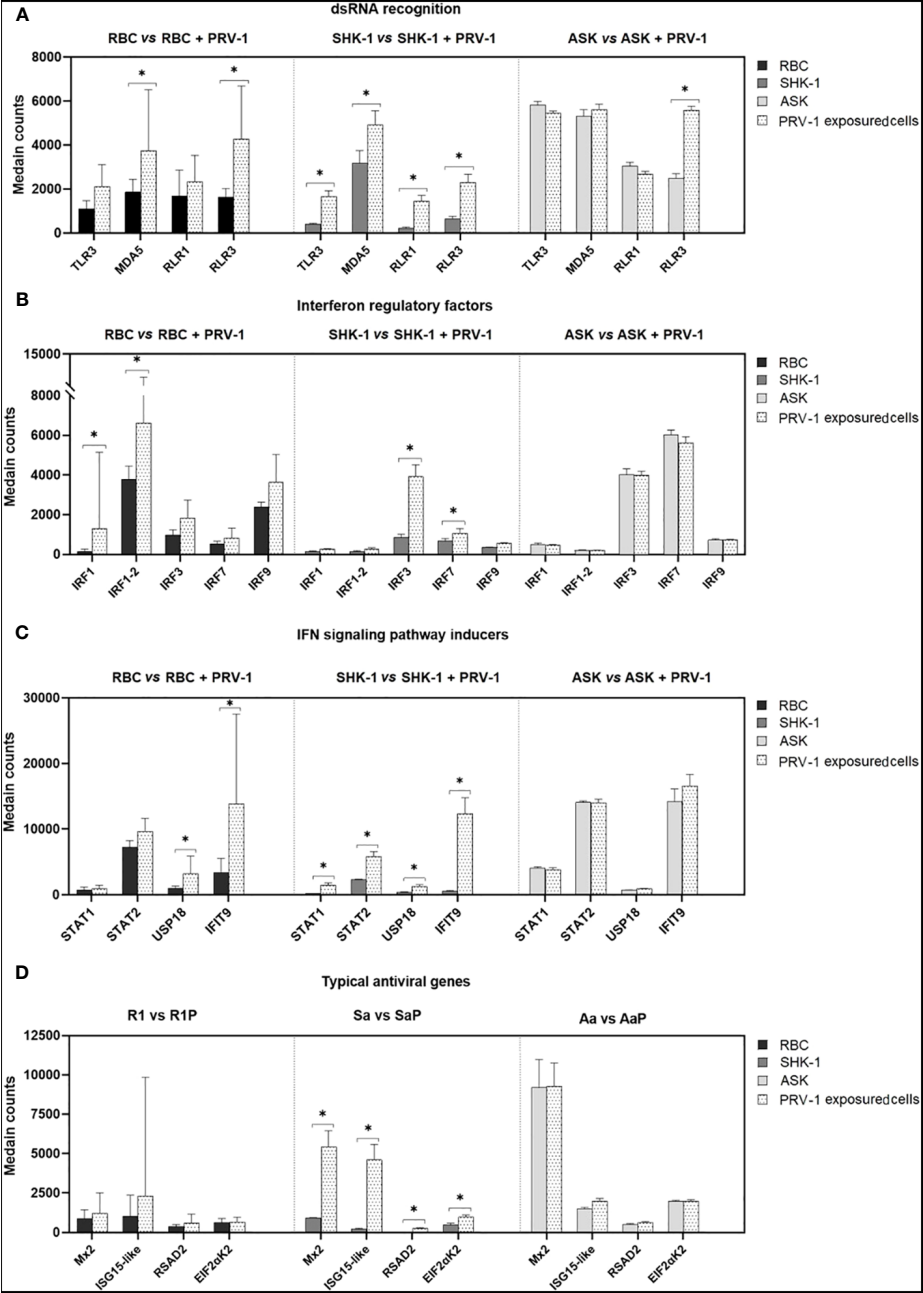
Comparison of the transcriptome responses linked to selected innate antiviral genes in RBC, SHK-1 and ASK exposed to PRV-1 (RBC vs RBC + PRV-1, SHK-1 vs SHK-1 + PRV-1 and ASK vs ASK + PRV-1, respectively). Regulation of **(A)** dsRNA pattern recognition receptors, **(B)** interferon regulatory factors, **(C)** genes involved in *IFN*-signaling patway activation and **(D)** IFN-inducible antiviral effectors. RBC vs PRV-1, n=6, SHK-1 vs PRV-1 and ASK vs PRV-1, n=3. *p<0.05.

Given the outcome of the differential expression analysis, GO and KEGG pathway enrichment analyses, 24 h exposure of RBC to PRV-1 triggered the activation of PRRs that recognize viral dsRNA (*MDA5* and *RLR3* induction) and signaling factors that regulate the secretion of IFNs and pro-inflammatory cytokines. To better understand the immune responses occurring in RBC after PRV-1 exposure, compared to non- susceptible kidney cell lines, we focused on genes typically involved in dsRNA viral recognition, signal transduction, *IFN*-pathway activation, and virus eradication. The comparison of the immune transcriptome responses of RBC to SHK-1 showed that SHK-1 respond more potently to PRV-1 than RBC by significantly inducing the expression of a wider repertoire of dsRNA pattern recognition receptors and typical antiviral genes. On the contrary, the comparison of RBC to ASK showed that ASK induced *RLR3*, while other typical antiviral responses were absent (**Figure 7**).

## 4 Discussion

The present transcriptional analysis showed that genes with the highest expression levels in RBC are primarily involved in respiratory processes, including multiple hemoglobins and mediators of heme biosynthesis. This is consistent with the traditional physiological characteristics of RBC as gas exchangers (3). Previous multi-omics analyses of salmonid RBC in response to viral infection revealed the expression of several genes involved in different aspects of immunity, including antigen presentation through MHC I and MHC II (8, 47). Current transcriptomic data indicated exceedingly high basal levels of the MHC I- associated protein- encoding genes, such as *UBA* and *UGA* genes, supporting A. salmon RBC role in innate immunity. Earlier characterization of *UBA* and *UGA* genes in rainbow trout leukocytes and lymphoid organs showed induced gene expression in response to viral infection (48). However, this was not the case in A. salmon RBC exposed to PRV-1 for 24 h, for which the short period of exposure to the virus may be a possible explanation.

The number of genes expressed in RBC in resting state was at comparable level as in ASK and SHK-1 cell lines, indicating that RBC are multifunctional. Although sets of genes involved in regulation of cellular homeostasis and survival (e.g. RNA processing and protein biosynthesis) showed similar expression patterns in all three cell types, genes associated with physiological functions which promote intracellular transport (e.g. endocytosis and nucleocytoplasmic transport) and molecule degradation (e.g. ubiquitin mediated proteolysis and autophagy) appeared to be more highly expressed in RBC. In contrast, genes essential for cellular structural integrity and differentiation, such as keratins (type I or II), serpines and cofilins, showed low transcription levels in RBC, while being more prominent in both kidney cell lines. Entry of PRV-1 into RBC have been predicted to occur via receptor- mediated endocytosis through *in silico* comparison of PRV proteins with MRV, for which viral uptake mechanisms are well characterized (49). In this sense, higher expression levels of genes involved in intracellular transport in RBC compared to non-susceptible ASK and SHK-1, may be linked to differences in uptake mechanisms. No genes involved in endocytic processes in RBC were significantly induced in response to 24 h-exposure to PRV-1.

Genes involved in signaling pathways triggered by viral invasion were expressed in RBC as well as ASK and SHK-1, confirming that RBC possess innate immune functions, as previously published (2, 5, 8, 47). Notably, the basal expression of genes associated with antiviral defense was more distinguished in RBC, compared to genes involved in responses to bacteria, indicating that RBC exhibit higher sensitivity to viruses.

Innate immunity represents the first line of host defense against invading pathogens, the recognition of which is mediated by PRRs (36, 50). Interestingly, RBC express a wide repertoire of PRRs, some of which have not been reported in salmonid erythrocytes earlier and that are able to detect pathogen-associated molecular patterns (PAMPs) derived from viruses and other pathogens. *TLR8* and *NLRC3- like* receptor genes appeared among the most highly expressed. Earlier studies on PRR signaling in fish showed that *TLR8* and *NLRC3-like* receptors trigger inflammatory responses through *MyD88*- and *NOD1/RIP2*- dependent signaling pathways upon recognition of synthetic ssRNA oligonucleotides and bacterial cell wall components, respectively (38, 42). The ability of salmonid RBC to manifest innate immune responses has most extensively been studied in response to RNA viruses (8, 47, 51) and there are few reports that demonstrate their immune responses to bacterial and parasites (51, 52). Although the gene expression of microbial- specific PRRs alone should not be considered indicative for their functional role, it may strengthen the notion of RBC as contributors to innate immunity against a broad range of infectious agents.

It is worth noting that various DEAD/H- box RNA helicases, recently characterized for their diverse roles in antiviral immunity in fish and mammals, were largely detected in RBC transcriptome (41, 53). Herein, *MDA5* and *RLR3* are reported in A. salmon RBC for the first time. Together with *RLR1*, these genes belong to the RLR family. Teleost RLRs, like in mammals, bind dsRNA viruses, and subsequently induce the activation of *type I IFN* signaling pathway and secretion of pro-inflammatory cytokines (53, 54). Previous transcriptional studies reported significant upregulation of *RLR1* in PRV-1 infected A. salmon, and *MDA5* and *RLR3* in viral hemorrhagic septicemia virus (VHSV) infected rainbow trout RBC (8, 22, 47).

RBC express multiple transcriptional activators that are essential for dsRNA-PRRs signaling, including several IRFs. For instance, binding of dsRNA to the cytosolic RNA sensors *RLR1* or *MDA5* leads to the activation of interferon promoter stimulating protein- 1 (*IPS* or *MAVS*). This activator, in association with TNF receptor- associated factor 3 (*TRAF3*) and TANK-binding kinase 1 (*TBK-1*), phosphorylates/activates *IRF3/7*, which potentiate the transcription of pro-inflammatory cytokines and IFNs (46, 53, 54). *TLR3*, similar to RLRs, is known to interact with TIR domain-containing adaptor (*TRIF* or *TICAM1*) to regulate the secretion of IFNs through the nuclear factor kappa B (*NF-κB*)- and *IRF3/7* - dependent signaling pathways (55). In general, secreted *IFN* and cytokines, in turn, bind to transmembrane *IFN*/cytokine receptors, and trigger the expression of IFN- stimulated genes by means of recruiting kinases and transcription factors, such as *JAK*, *STAT1/2*, *IRF9* and/or *IRF1* (53–55). The identification of genes corresponding to such complete signaling pathways in RBC transcriptome not only reinforces RBC characterization as immune mediators, but also contributes to our original hypothesis that they regulate multiple immune functions through both well characterized and unexplored signaling pathways in salmonid RBC.

A rather intriguing finding was the expression of several interleukin (IL) and chemokine receptors in A. salmon RBC, but only a few of the corresponding cytokines were expressed. As in mammals, fish cytokines are secreted by many cell types and involved in cell-to-cell communication though an endocrine and/or paracrine manner (56, 57). The expression of pro-inflammatory IL receptor subunits, such as *IL6R* and *IL1R*, may imply immune activation of RBC upon binding to *IL1* and *IL6*, secreted by other immune cells. Fish and mammalian *IL10* and *IL10R* regulate anti- inflammatory functions, a feature that suggests involvement in mechanisms of viral persistence (58). Since RBC express *IL10R*, they may participate in processes related to such mechanism, for example in the persistent phase of PRV-1 infection (59). In contrast to rainbow trout RBC, which were shown to express *IL1β*, *IL8* and *IFNγ* in response to infectious hematopoietic necrosis virus (IHNV) and thermal stress, A. salmon RBC demonstrated high transcript levels of only *IL15* and *IL34* (7, 36). Studies on the characterization of *IL15* in rainbow trout suggested its involvement in CD4+ T cell survival, where it induces *IFNγ* through a *STAT5p*- dependent signaling pathway (60, 61). The function of *IL34* is modestly explored in salmonids. However, in recent studies in fresh water fish species such as Largemouth bass (*Micropterus salmoides*) and grass carp (*Ctenopharyngodon idella*), *IL34* was suggested to be involved in macrophage activation (62, 63).

The comparison of RBC to ASK and SHK-1 revealed sets of genes, which were exclusively expressed in RBC, and involved in innate/adaptive immune processes and chemotaxis. This supports the multifunctional nature of RBC, while providing insight into their unique immunological features. Indicatively, among the wide assortment of IRFs identified in the total transcriptome, *IRF4* expression appeared only in RBC. Earlier characterization of IRFs in A. salmon showed that *IRF4*, similar to its mammalian counterpart, inhibits *IFN* production (64). Additional immunosuppressive effects on RBC may be mediated by *IL1R2*, which have been shown to compete with *IL1* for binding *IL1Ra* in seabream (*Sparus aurata*) and grass carp (*Ctenopharyngodon idellus*) (65, 66). In mammals, *CCR9* is distributed on the surface of intestine cells where it binds its specific ligand *CCL25*. In both mammals and teleost, upregulation of *CCL25* in gut has been associated with infiltration of *CCR9*- expressing inflammatory cells (67, 68). The expression of *CCR9* in RBC may indicate that, similarly to immature T-lymphocytes, they may migrate into tissues expressing *CCL25* ligand. Mammalian C-C chemokine 4 (*CCL4*) is commonly expressed in different antigen- presenting cells (APC), and *CCL4* regulation has only recently been studied in fish (57, 69, 70). Functional characterization of *CCL4* in orange- spotted grouper (*Epinephelus coioides*) showed that recombinant *CCL4* exhibits chemotactic activity, attracting leukocytes, such as macrophages and NK-cells, and stimulating lymphocyte differentiation (71); thus, the role of *CCL4* was suggested to be conserved in teleost and mammals (69, 71). Since A. salmon RBC express *CCL4*, they may be involved in inflammatory responses by recruiting macrophages and NK-cells and/or triggering lymphocyte differentiation. Although, it is hard to assume the role and involvement of these cytokines and cytokine receptors in the immune functions of RBC, hypotheses regarding the possible migration of RBC into inflammatory tissue like other circulating immune cells could represent an open and interesting field of study.

A few immune genes were significantly induced in RBC 24 h after PRV-1 encounter. Most DEGs are involved in dsRNA recognition and subsequent signal transduction via IRFs, but *type I IFN* and IFN- stimulated genes were not found induced. In contrast, several genes implicated in RNA virus recognition and antiviral defense were significantly expressed in SHK-1, while remaining at basal levels in ASK. Previous transcriptional analysis of ASK cells in response to synthetic dsRNA analogue, poly(I:C), revealed significant induction of the RNA-specific PRRs genes *MDA5* and *RLR3*, and antiviral effectors genes, such as *type I IFN* and *Mx1* and *ISG15*, 12 h post stimulation (72). This suggests that despite the ability of ASK to respond to naked dsRNA, the processes associated with ligand recognition and initiation of immune defense may differ in response to purified virus. To date, recognition of PRV-1 in RBC has primarily been associated with the induction of endosomal *TLR3* and cytosolic *RLR1* (6, 8). Our data, however, showed upregulation of the *MDA5* and *RLR1* genes after 24 h-exposure to the virus. Several putative PRR- genes for RNA viruses were significantly induced in SHK-1, including *TLR3*, *RLR1*, *MDA5* and *RLR3*, whereas only *RLR3* was induced in ASK. PRV-1 propagation is not supported by SHK-1, and the upregulation of many genes involved in a range of different antiviral pathways in response to virus, may be a possible explanation. Interestingly, the *RLR3* gene was significantly induced in all PRV-exposed cells. In contrast to *RLR1* and *MDA5*, the role of *RLR3* in antiviral immunity in fish cells is poorly understood. In mammals, *RLR3* is associated with both positive and negative contribution to antiviral signaling in a concentration- dependent manner. *RLR3*, when at low levels, functions synergistically with *MDA5*, and thereby enhance *MDA5*-mediated antiviral signaling. Oppositely, *RLR3* at high expression levels competes with *RLR1* and *MDA5* for dsRNA viral recognition and suppresses RLR signaling pathway by inhibiting receptor interaction with the *IPS* activator (73). In teleost, *RLR3* has mainly been associated with positive regulation of antiviral signaling; its expression was linked to significant induction of antiviral effectors, such as *Mx*, in rainbow trout, and decrease of grass carp reovirus (GCRV) and spring viremia of carp virus (SVCV) titers in black carp (*Mylopharyngodon Piceus*) *in vitro* (40, 74). In contrast to mammals, functional characterization of *RLR3* in fish did not show suppression or synergy with *MDA5*, but rather a parallel function (40). Relative expression of *RLR3* in A. salmon RBC, ASK and SHK-1 in response to PRV-1 do not provide sufficient evidence for its putative function. However, its significant induction may indicate a pivotal contribution to viral recognition and the following antiviral events in the cell.

As mentioned above, *MDA5* activation is commonly followed by the transcriptional activity of *IRF3* and/or *IRF7* (53, 75). Induction of these IRF genes has previously been reported in salmonid erythrocytes at later stages of PRV-1 infection *in vivo* (8). Here, only *IRF1* was significantly upregulated, whereas there was not significant induction of *IRF3* and *IRF7* in response to PRV-1. *IRF1* has been shown to actively participate in induction of IFN and ISG transcription as a response to RNA viruses in mammals and fish (46, 76). As opposed to RBC, ASK and SHK-1 expressed low levels of *IRF1* both pre- and post- exposure to PRV-1. In contrast, the expression of *IRF3* and *IRF7* was significantly induced in SHK-1 after PRV-1 exposure, while expressed at constitutively high levels in ASK. Previous investigation of IRF involvement in antiviral defense in mammals revealed that *IRF1* may function independently of *IRF3/IRF7* (77). Considering that RBC is the only cell type susceptible to PRV-1, the low activation of *IRF3/7* and strong induction of *IRF1* in RBC could represent a difference associated with antiviral responsiveness to PRV-1.

The entry of PRV into RBC likely occur through endosomal uptake, as its mammalian counterpart MRV (78). This process leads to virion disassembly at late endosomes and release of transcriptionally active viral core particles into the cytoplasm that subsequently produce capped, but not poly- adenylated ssRNA copies (79). Interferon-induced protein with tetratricopeptide repeats 5-like (*IFIT9*, also referred to as IFIT5 in rainbow trout) and ubl carboxyl- terminal hydrolase 18-like (*USP18*) have been implicated in inhibition of VHSV replication and negative regulation of immune responses mediated by type I IFN, respectively (80, 81). Both *IFIT9* and *USP18* were significantly upregulated in RBC, which in correlation with the expression profile of PRRs and IRFs, may be indicative of viral status in the cells. Complementary to this, no induction of typical antiviral genes, such as *Mx*, interferon-stimulated gene 15- like (*UBIL*), *PKR* (referred to as *EIF2aK2*) and viperin-like (*RSAD2*), which have previously been found upregulated in PRV-1 infected RBC *in vivo*, was observed after a 24 h viral stimulation of RBC (8). In contrast, SHK-1 responded to PRV-1 by inducing the expression of several IFN-inducible genes and their corresponding transcription factors (e.g. *STAT1/2* that regulates *Mx* and *ISG15* transcription) significantly. Typical antiviral response genes highly expressed in SHK-1 but not in RBC, such as *Mx2* and *ISG15-like*, may play a role in the successful eradication of the virus (72, 80, 82, 83). The comparison, however, of RBC to ASK showed that no typical antiviral responses were observed in ASK. Instead, pro-inflammatory cytokines *IL-11* and *CXCL10* were significantly induced. These findings may indicate that ASK cells lack viral uptake and sufficient sensing of viral RNA, whereas SHK-1 cells may take up PRV, respond, but inhibit viral replication more efficiently by strong antiviral responses. The antiviral response in RBC may be delayed compared to the SHK-1 response, which might favor the replication of the virus.

In conclusion, the present transcriptional analysis supports previous characterization of RBC as multifunctional cells with both physiological and immunological properties. In contrast to ASK and SHK-1 cells, RBC showed higher expression levels of genes related to endocytosis and intracellular transport and uniquely expressed *CCL4* and *CCR9* genes, suggesting putative chemotactic activity and an ability to recruit immune cells. Exposure of RBC to PRV-1 for 24 h induced a typical antiviral response of intermediate strength, stronger than in ASK cells, but possibly delayed compared to responses in SHK-1. A difference in IRF gene induction (*IRF1* in RBC, *IRF3/7* in SHK-1 cells) may affect the antiviral response pathway and allow onset of PRV-1 replication in RBC.

## Data availability statement

The original contributions presented in the study are publicly available. This data can be found here: NCBI SRA BioProject- PRJNA1028935, https://www.ncbi.nlm.nih.gov/bioproject/PRJNA1028935.

## Ethics statement

The animal study was approved by Norwegian Animal Research Authority/Norwegian University of Life Sciences Aquatic facility. The study was conducted in accordance with the local legislation and institutional requirements.

## Author contributions

TT: Data curation, Formal Analysis, Investigation, Methodology, Validation, Visualization, Writing – original draft, Writing – review & editing, Software. AYMS: Data curation, Formal Analysis, Methodology, Supervision, Validation, Visualization, Writing – original draft, Writing – review & editing, Software. SB: Formal Analysis, Methodology, Validation, Writing – review & editing. JBJ: Investigation, Supervision, Validation, Visualization, Writing – review & editing. ER: Conceptualization, Funding acquisition, Resources, Supervision, Validation, Writing – review & editing. ØW: Conceptualization, Formal Analysis, Funding acquisition, Investigation, Methodology, Project administration, Resources, Supervision, Validation, Writing – review & editing. MKD: Conceptualization, Data curation, Formal Analysis, Funding acquisition, Investigation, Methodology, Project administration, Resources, Supervision, Validation, Writing – original draft, Writing – review & editing.
