# Supplementary material for: Transcriptomics of early responses to purified Piscine orthoreovirus-1 in Atlantic salmon (Salmo salar L.) red blood cells compared to non-susceptible cell lines
Source: Front Immunol. 2024 Feb 14;15:1359552. doi: 10.3389/fimmu.2024.1359552 (PMC10899339; doi:10.3389/fimmu.2024.1359552)
Supplement: Supplementary file 1 [file DataSheet_1.docx]

Supplementary material A

Table 1. Total sequenced reads and alignment rate of mapping.

| Group | Sample ID | Total clean reads | Reads aligned to genome % | Reads aligned to exons/genes % |
| --- | --- | --- | --- | --- |
| Control RBC | R1 | 53658189 | 85.58 | 62 |
|  | R2 | 40108398 | 90.41 | 71.9 |
|  | R3 | 51639660 | 90.05 | 69.5 |
|  | R4 | 42767611 | 88.69 | 63.8 |
|  | R5 | 48615954 | 88.27 | 65.6 |
|  | R6 | 47435421 | 89.2 | 65.8 |
| Control ASK | A1 | 53246205 | 89.32 | 72.2 |
|  | A2 | 45226606 | 91.01 | 76.8 |
|  | A3 | 54329721 | 90.83 | 73.4 |
| Control SHK-1 | S1 | 38996895 | 91.07 | 74.9 |
|  | S2 | 37103132 | 91.65 | 70.5 |
|  | S3 | 52825689 | 91.7 | 77.6 |
| RBC and PRV-1 | R1P | 47754341 | 88.91 | 62.4 |
|  | R2P | 20422945 | 90.32 | 69.1 |
|  | R3P | 54396644 | 90.18 | 68.4 |
|  | R4P | 49856293 | 86.08 | 62.5 |
|  | R5P | 44057896 | 80.37 | 71.9 |
|  | R6P | 43903474 | 87.15 | 65 |
| ASK and PRV-1 | A1P | 55711603 | 92.33 | 75 |
|  | A2P | 53796949 | 91.72 | 76.1 |
|  | A3P | 50850763 | 91.79 | 74.8 |
| SHK-1 and PRV-1 | S1P | 49158746 | 91.96 | 73.8 |
|  | S2P | 38503460 | 91.87 | 72.3 |
|  | S3P | 48838081 | 92.11 | 77.2 |


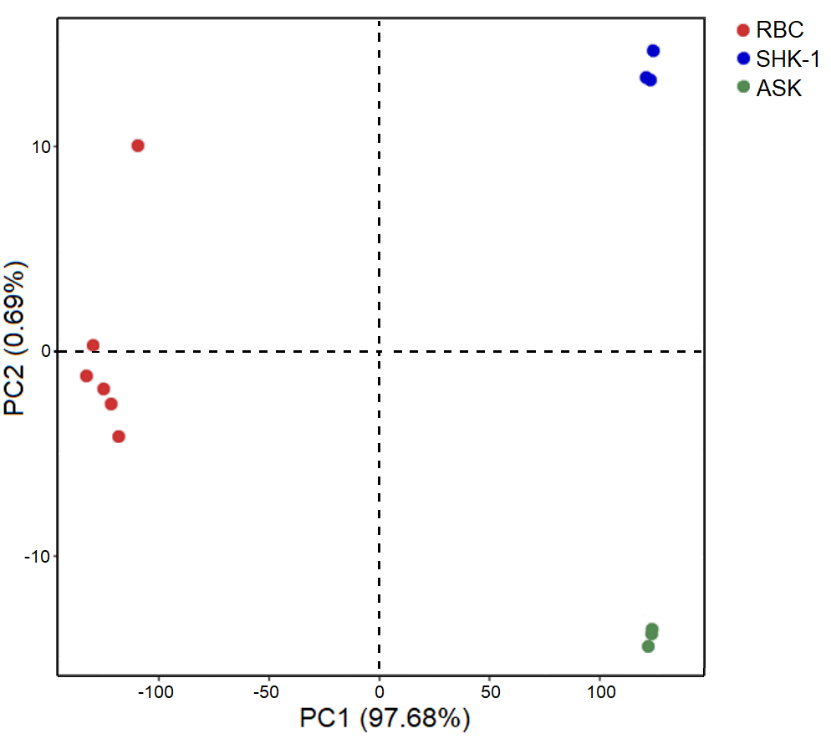


Figure 1. Principal component analysis (PCA) for Atlantic salmon RBCs (red), ASK (green) and SHK-1 (blue) gene set of the analysis with percentages of variance associated with each axis.


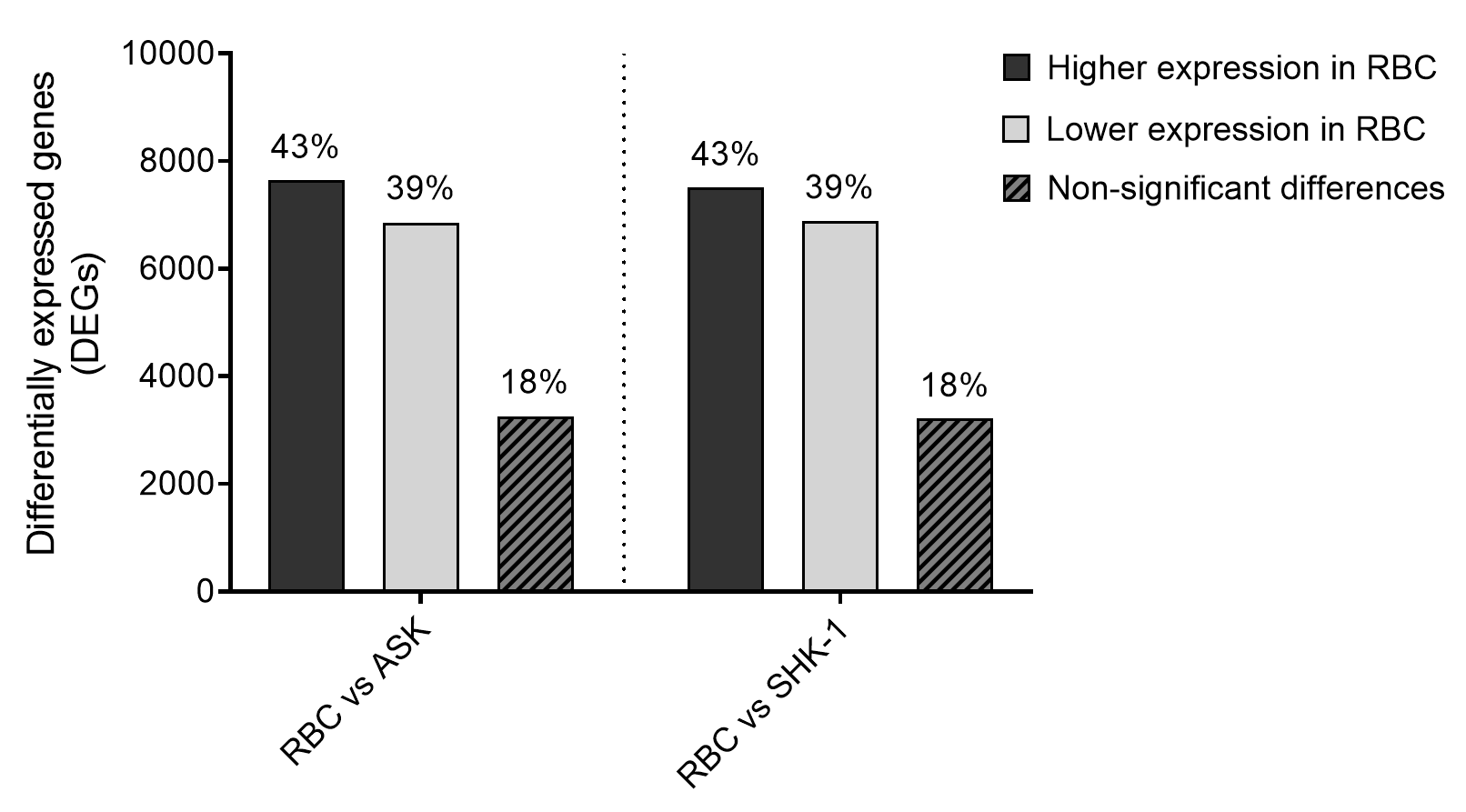


Figure 2. Differential gene expression analysis of A. salmon RBC vs ASK and SHK-1 in resting state. In each comparison, the percentage of genes with higher expression in RBC, lower expression in RBC, and no significant differences between the cell lines are provided above each bar graph. Cutoff ≥ 10 normalized median counts.


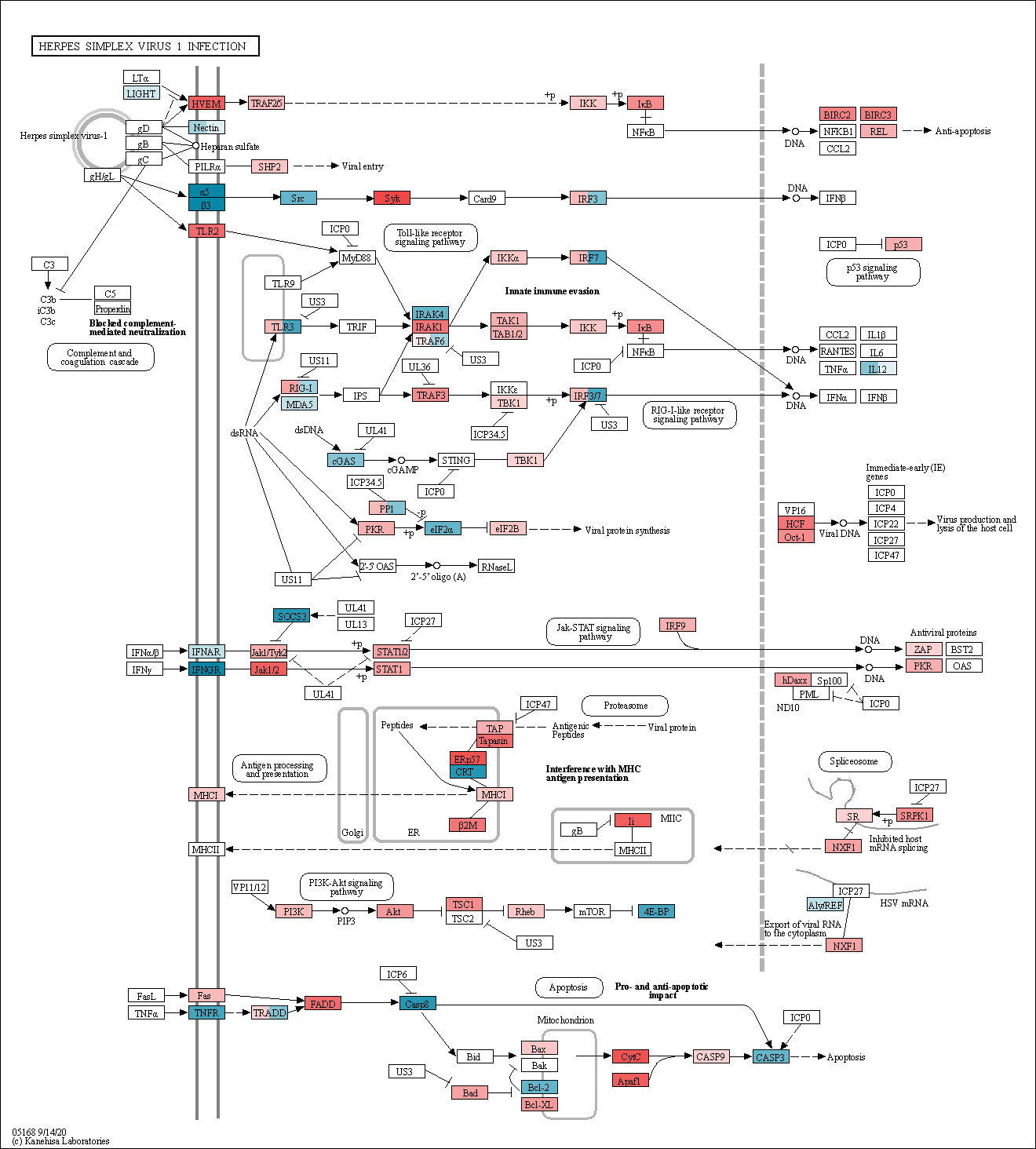


Figur 3. “Herpes simplex virus 1 infection”- sasa05168 pathway in Kyoto Encyclopedia of Genes and Genomes (KEGG) by Kanehisa Laboratories. Genes colored red showed significantly higher transcripts in RBC, and genes colored cyan showed similar and significantly higher transcripts in ASK and SHK-1 (thus lower transcripts in RBC). Genes with significantly different expression levels between ASK and SHK-1, and also with RBC were colored red and cyan. The higher the significant differences the darker the color of the genes.


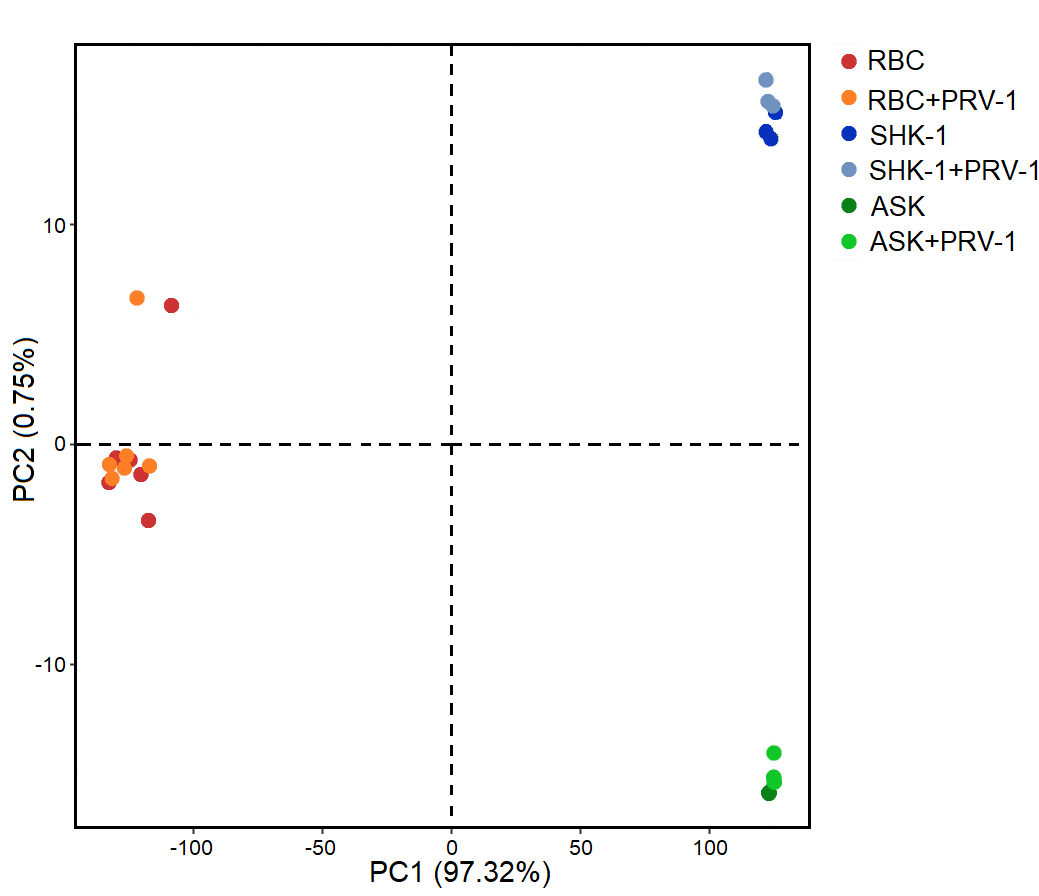


Figure 4. Principal component analysis (PCA) for Atlantic salmon RBCs, ASK and SHK-1 exposed to PRV-1 and the unexposed controls, with percentages of variance associated with each axis.
